# Angiotensin II Induces Abdominal Aortic Branch Aneurysms in *Fibrillin-1* ^*C1041G/+*^ Mice

**DOI:** 10.1101/2025.11.26.690689

**Published:** 2025-11-30

**Authors:** Michael K. Franklin, Deborah A. Howatt, Jessica J. Moorleghen, Mary B. Sheppard, Hisashi Sawada, Hong S. Lu, Alan Daugherty

## Abstract

**Background:**

Mice harboring a missense variant (C1041G) of *fibrillin-1* ( *Fbn1* ) have been used extensively for aortopathy research, but do not mimic all facets of the human disease. The role of increased angiotensin II (AngII) or blood pressure in determining the arterial phenotype of these mice remains incompletely defined. The purpose of this study was to define whether AngII, directly or via increased blood pressure, promoted aortic disease in the proximal thoracic aorta and beyond.

**Methods:**

*Fbn1* ^*+/+*^ *and Fbn1* ^C1041G/+^ littermates were infused with either AngII or norepinephrine (NE) via subcutaneously implanted osmotic pumps. Aortic dimensions were determined using in situ imaging. Micro Computed tomography (microCT) was used to determine the localization of aortic pathologies.

**Results:**

AngII infusion dramatically augmented aortopathy in *Fbn1* ^C1041G/+^ mice. Aortic dissection was visible within 3 days of AngII infusion. Over 50% of male *Fbn1* ^C1041G/+^ mice died during AngII infusion, primarily due to aortic rupture in either the thoracic or abdominal regions. Surviving males had greatly increased ascending aortic diameters and the appearance of pathology at branches of the abdominal aorta. Female mice had a much lower incidence of death but had greatly increased aortic diameters. Although NE infusion also increased systolic blood pressure, it did not significantly augment mortality or aortic diameters. MicroCT discerned novel pathology during AngII infusion that included development of branch aneurysms in the celiac and superior mesenteric arteries.

**Conclusion:**

AngII greatly enhanced aortic pathology in *Fbn1* ^C1041G/+^ mice, manifested by aortic rupture in both the thoracic and abdominal regions and development of pathologies at aortic branches of the celiac and superior mesenteric arteries.

**HIGHLIGHTS:**

- Aortic branch aneurysms have recently been demonstrated to be prominent in Marfan patients but have not been reported in Marfan mouse models.
- Angiotensin II infusion into *fibrillin-1* ^*C1041G/+*^ mice promoted development of prominent aortic pathologies, including enhanced aneurysm and rupture in both the thoracic and abdominal regions.
- Angiotensin II infusion into *fibrillin-1* ^*C1041G/+*^ mice promoted development of pronounced aneurysms at the aortic branches of the celiac and superior mesenteric arteries.

## INTRODUCTION

Fibrillin-1 (FBN1) is a large protein (~350 kDa) that is hypothesized to be required for development of a scaffold on which elastic fibers are developed. Genetic variants of *FBN1* are the underlying cause of Marfan syndrome. The manifestations of Marfan Syndrome are highly diverse, which may be at least partially attributable to the fact that there are over 2,000 variants.[1] While attention has commonly focused on the aberrant dilation of the aortic root in Marfan patients, it is increasingly recognized that these individuals have diverse vascular pathologies. These include aneurysm, dissection, and rupture in aortic areas beyond the root, including the ascending, descending thoracic, and abdominal regions.[2] Aneurysms also frequently occur at multiple aortic branch points. This includes aortic branches arising from the aortic arch (brachiocephalic, carotid, subclavian), descending thoracic (vertebral, bronchial), suprarenal (celiac, superior mesenteric, renal), and infrarenal regions (iliac).[3] The presence of ancillary aneurysms further increased the complexity of pathologies induced by *FBN1* variants.

While *FBN1* variants cause a diversity of vascular phenotypes, experimental models using *FBN1* variants are predominantly focused on the proximal thoracic aorta.[4] The most commonly used model is mice expressing a *Fbn1*$^{C1041G/+}$ variant.[5] These mice have a modest expansion of the aortic root and ascending aorta,[6, 7] which exhibits profound sexual dimorphism, with progressive enlargement only occurring in male mice up to one year of age.[7, 8] There have been no reports of aortic dissection or rupture in *Fbn1* $^{C1041G/+}$ mice.

To determine the role of angiotensin II (AngII) in arterial diseases, many studies have employed chronic subcutaneous infusions, most commonly at a rate of 1,000 ng/kg/min.[9] This procedure in wild type mice promotes perivascular fibrosis in many arterial beds.[10] When combined with hyperlipidemia and BAPN administration, AngII infusion promotes localized pathologies with augmentation of proximal thoracic aortopathy and progressive expansion and rupture of the suprarenal aorta.[11-13] AngII has been infused into *Fbn1*$^{C1041G/+}$ mice. This includes very high infusion rates of AngII (4.5 mg/kg/d = 3,125 ng/kg/min) that increased ascending aorta diameters, with some studies having all mice die of ascending aortic rupture within 4 weeks of infusion.[14-16] AngII infusion at 1,000 ng/kg/min in *Fbn1* $^{C1041G/+}$ mice in normolipidemic background[17] or deficient in ApoE also resulted in increased aortic aneurysms in both the ascending and abdominal regions.[18]

The role of increased AngII activation in aortic pathology has not been explored extensively in *Fbn1*$^{C1041G/+}$ mice. Therefore, this study was designed to address the following questions: 1. Whether the sexual dimorphism of vascular diseases that occur in *Fbn1* $^{C1041G/+}$ mice is retained under AngII activation. 2. Whether pathologies develop beyond the proximal thoracic aorta in a mode that recapitulates the human disease. 3. Whether blood pressure per se augments vascular diseases or whether the effect is specific to AngII activation.

## METHODS

### Data Availability

Detailed materials and methods are available in this manuscript. Numerical data are available in the Supplemental Excel File.

### Mice

Studies were performed in accordance with recommendations for design and reporting of animal aortopathy studies.[19] All experiments used littermate controls. *Fbn1*$^{C1041G/+}$ (stock #012885) mice were obtained from The Jackson Laboratory as described previously.[8] Male and female *Fbn1*$^{C1041G/+}$ mice were bred to C57BL/6J female and male mice, respectively, to generate male and female *Fbn1*$^{+/+}$ and *Fbn1*$^{C1041G/+}$ littermates. Littermates were separated by sex and randomly assigned to housing groups after weaning. Mice were housed up to 5 per cage, maintained on a 14-hour light/10-hour dark cycle, fed Teklad Irradiated Global 18% Protein Rodent Diet # 2918 ad libitum, and allowed ad libitum access to water via a Lixit system. Bedding was provided by P.J. Murphy (Coarse SaniChip) and changed weekly during the study. Cotton pads were provided as enrichment. The room temperature was maintained at 21-23 °C, and the humidity was maintained at ~ 50%.

Both male and female mice were studied. All experiments were approved by the University of Kentucky IACUC (Protocol # 2018-2967).

### Genotyping

Mice were genotyped after weaning and termination, respectively, using tail tissue: group allocation was based on genotyping performed after weaning at postnatal day 28, and the genotype was confirmed again using tissue acquired at the termination of each study. *Fbn1*$^{C1041G/+}$ was assayed using forward primer (5'-CTCATCATTTTTGGCCAGTTG-3') and reverse primer (5'-GCACTTGATGCA CATTCA CA-3') covering a loxP-flanked neomycin resistance cassette placed in intron 24, which is not present in wild type mice. The protocol used was as described by The Jackson Laboratory. *Fbn1*$^{+/+}$ generates a 164 bp product. *Fbn1*$^{C1041G/+}$ generates a 212 bp product. Post-termination validation genotyping was performed by Transnetyx in a blinded manner.

## Subcutaneous Infusions

After random assignment, AngII (1,000 ng/kg/min dissolved in saline; Product # 4006473; CAS # 4474-91-3; Bachem) or its vehicle (saline), or norepinephrine (NE 5.6 mg/kg/day; dissolved in saline containing 0.2% wt/vol ascorbic acid[20]) or its vehicle (0.2% wt/vol ascorbic acid in saline) was infused through a subcutaneously implanted osmotic pump (ALZET LLC). Alzet model 2001 was used for 3 days and model 1004 for 28 days of infusion, respectively, in mice at 10 to 14 weeks of age.[21] Surgical staples used to close incision sites were removed 7-10 days after surgery. Postoperative pain was alleviated by application of a topical lidocaine cream (4% wt/wt; Cat # 59-930, HealthWise).

## Systolic Blood Pressure Measurements

Systolic blood pressure was measured on conscious mice by a non-invasive tail-cuff system (MC4000 Multi Channel system, Hatteras Instruments) following our standard protocol.[22] Data were collected at the same time each day for 3 consecutive days. Criteria for accepted data were systolic blood pressure between 70 and 200 mmHg and standard deviation < 30 mmHg for at least 5 successfully recorded data/mouse/day. The mean systolic blood pressure of each mouse from the 3-day measurements was used for data analysis.

## Necropsy

All study mice were checked at least once every day. Necropsies were performed immediately to determine the cause of death after carcasses were found. Aortic rupture was defined as the presence of extravascular blood that accumulated in the body cavity. The location of blood egress was determined by the location of the blood clot and a discernible disruption of the aortic wall.

## Micro Computed Tomography (microCT)

MicroCT was performed as described previously.[23] Mice were euthanized by an overdose of ketamine and xylazine cocktail (90 and 10 mg/kg, respectively). The thoracic cavity was cut open, and the right atrium was nicked to allow the exit of blood flow. Saline (5 ml) was perfused through the left ventricle. The right atrium was sealed using superglue immediately after perfusion, and Microfil® (Flow Tech, Inc.) was injected through the same catheter. Once Microfil was visualized in the arterioles surrounding the small intestine, the catheter was clamped shut to prevent backflow of Microfil® into the thoracic cavity, and the animal was set aside to allow the compound to harden for ~90 minutes.

After Microfil perfusion, animals were scanned using a Skyscan 1276 MicroCT (Bruker) or a U-CT Optical Imaging system (MILabs, Netherlands). CT-scanned images were reconstructed using the N-Recon program (Bruker, Belgium) or MILabs reconstruction program (MILabs, Netherlands) to adjust for beam hardening and ring artifacts. 3D reconstruction and measurements were performed using the 3D slicer program. All bones and vasculatures not of interest were removed using a scissor tool within the program to display the aorta and its major branches. To visualize branch arteries, all the other surrounding vasculatures were removed using the scissor tool.

## Measurement of in situ Aortic Diameters

Mice were terminated by overdose of ketamine and xylazine mixture followed by cardiac puncture and saline perfusion. The order in which mice were terminated was randomized. Aortas were dissected away from the surrounding tissue. A black plastic sheet was inserted beneath the aorta and heart to increase contrast and facilitate visualization of aortic borders.[24] Optimal Cutting Temperature compound (Sakura Finetek) was introduced into the left ventricle to maintain aortic patency before imaging. Aortas were imaged using a Nikon SMZ25 stereoscope, and measurements were recorded using NIS-Elements AR 5.11.03 software (Nikon Instruments Inc.). Ascending aortic diameters were measured at the largest width perpendicular to the vessel.

## Pathology

Thoracic aortas with major branches were harvested from surviving mice after 3 days of AngII infusion and immersed in neutrally buffered formalin (10% wt/vol). Aortas were embedded in OCT and sectioned with a cryostat.

Abdominal aortas with major branches were dissected free and immersed in neutrally buffered formalin (10% wt/vol) overnight, followed by a series of dehydration steps in increasing concentrations of ethanol. The aortas were then embedded in paraffin wax. Tissue sections (5 µm) were collected with a microtome. Paraffin-embedded sections (5 µm) were deparaffinized using limonene (Cat # 6533A, Medical Chemical Corporation).

Hematoxylin and eosin (H&E, Cat # 26043-06, Electron Microscopy Sciences; Cat # AB246824, abcam) and Verhoeff iron hematoxylin staining were performed, respectively, to visualize pathologies and elastic fibers. Picrosirius red/methyl green staining was performed to visualize collagen fibers. Immunostaining was performed using primary and secondary antibodies listed in the Supplemental Materials Major Resources Tables. NovaRed (Cat #SK-4805, Vector) was used as chromogen. Images of histological staining and immunostaining were captured using an Axioscan 7 (Zeiss) or Nikon Eclipse N*i* and imaged using ZEN v3.1 blue edition (Zeiss) or NIS-Elements AR 5.11.03 software (Nikon Instruments Inc)

## Statistical Analyses

SigmaPlot version 15 or 16 (SYSTAT Software Inc.) was used for statistical analysis. Data were presented as either mean ± SEM or median with the 25$^{th}$ and 75$^{th}$ percentiles, depending on whether the data were analyzed by parametric or nonparametric tests. Normality and homogeneous variance assumptions for data with n≥6/group were assessed using the Shapiro-Wilk test and the Brown-Forsythe test, respectively. Student's t-test was used for the data that met both normality and homoscedasticity to compare means. For data that did not pass either the normality or equal variance test or n<6/group, Mann-Whitney U-test was applied. Statistical significance was set at P<0.05.

## RESULTS

### AngII Infusion Reduced Survival in Male *Fbn1*$^{C1041G/+}$ Mice

Infusion of AngII for 3 days led to distinctive blood accumulation in both the ascending and descending thoracic aorta of *Fbn1*$^{C1041G/+}$, but not *Fbn1*$^{+/+}$, mice. This phenotype was restricted to the outer margins of the media (Figure S1), as has been demonstrated in several other modes of inducing thoracic aortic pathology in mice.[21, 25]

AngII infusion into male *Fbn1*$^{C1041G/+}$ mice for 28 days led to a high incidence of death (~65%) that was significantly higher compared to the mortality rate in *Fbn1*$^{+/+}$ mice (Figure 1A). Necropsies of all mice that died within 14 days of AngII infusion revealed characteristic blood accumulation in either the thoracic or abdominal cavity, with approximately equivalent incidence. Since the precise loci of blood egress could not be reliably identified, rupture sites were categorized broadly as thoracic or abdominal (Figure 1B). All the deaths due to abdominal aortic rupture occurred within the initial 7 days of AngII infusion. Deaths due to thoracic aortic rupture were also prominent within the first 7 days of AngII infusion, but some occurred during more protracted infusion. Some deaths occurred late during AngII infusion that were not discernible as being due to loss of vascular integrity (Supplemental Table 1). In contrast to the high mortality observed in male mice, female *Fbn1*$^{C1041G/+}$ mice exhibited a low incidence (20%) of death during AngII infusion, which did not differ significantly from that of *Fbn1*$^{+/+}$ mice (Figure 1C). All the vascular deaths in females were attributed to thoracic aortic rupture (Figure 1D).

Aortas were visualized in mice that survived 28 days of AngII infusion, and in situ diameters were measured as described previously.[24] Aortas of both *Fbn1*$^{+/+}$ and *Fbn1*$^{C1041G/+}$ male mice infused with saline had a similar opacity, which was increased following AngII infusion (Figure 1E). AngII infusion significantly increased aortic diameters in both *Fbn1*$^{+/+}$ and *Fbn1*$^{C1041G/+}$ male mice (Figure 1F). Female *Fbn1*$^{+/+}$ and *Fbn1*$^{C1041G/+}$ mice also had similar opacity during saline infusion (Figure 1G). AngII infusion into *Fbn1*$^{+/+}$ female mice failed to promote an overt change in opacity but diameters were increased modestly. AngII infusion into female *Fbn1*$^{C1041G/+}$ mice promoted grossly discernible pathology in the ascending aorta and pronounced increases in diameters (Figure 1H).

Aortas were dissected free to determine whether pathologies were present in any other regions of the aorta. During this tissue processing, longitudinal tortuosity in *Fbn1*$^{C1041G/+}$ mice infused with AngII was noted. Therefore, aortic lengths were measured from the branch of the subclavian to the iliac bifurcation. AngII infusion significantly increased the length of the aorta, irrespective of *Fbn1* genotype (Figure 1K). The length of the aorta was much greater in male *Fbn1*$^{C1041G/+}$ mice and was significantly increased during AngII infusion (Figure 1K). In contrast, the aortic length was not different between saline and AngII infusion in female *Fbn1*$^{C1041G/+}$ mice.

## Norepinephrine Increased Blood Pressure but Failed to Induce Pronounced Aortic Disease

To determine whether increased systolic blood pressure induced by AngII infusion augmented aortic pathologies in *Fbn1*$^{C1041G/+}$ mice, this strain was infused with NE. Infusion of NE at a rate of 5.6 mg/kg/day led to significant increases in systolic blood pressure in both male and female *Fbn1*$^{+/+}$ and *Fbn1*$^{C1041G/+}$ mice (Figure S2A and B). In contrast to AngII infusion, there were no deaths in either male or female *Fbn1*$^{+/+}$ or *Fbn1*$^{C1041G/+}$ mice during NE infusion (Figure S2C and D). Also, there were no apparent changes in the in situ appearance of aortas during NE infusion in either male or female *Fbn1*$^{+/+}$ and *Fbn1*$^{C1041G/+}$ mice (Figure S2E and G), nor in the maximum external diameter of the ascending aorta (Figure S2F and H). Furthermore, the increased systolic blood pressure induced by NE infusion failed to alter aortic length or develop any grossly apparent aortic pathologies (Figure S2I-L).

## AngII Promoted Development of Abdominal Aortic Branch Aneurysms in *Fbn1*$^{C1041G/+}$ Mice

Ex vivo examination of aortas revealed the development of aortic branch aneurysms in the abdominal region of AngII-infused *Fbn1*$^{C1041G/+}$ mice. To gain further insight, aortic imaging was performed using microCT in groups of saline and AngII-infused mice, respectively. No abdominal aortic branch aneurysms were detected in male and female *Fbn1*$^{+/+}$ and *Fbn1*$^{C1041G/+}$ mice following infusion with saline, or in *Fbn1*$^{+/+}$ male and female mice infused with AngII (Figure 2A and S3A). In contrast, AngII infusion into *Fbn1*$^{C1041G/+}$ male and female mice produced marked aneurysmal expansion in the celiac and superior mesenteric arteries, while not affecting the left or right renal arteries (Figure 2B and S3B). The increased aneurysm at the aortic branches of the celiac and superior mesenteric arteries was a highly consistent pathology in *Fbn1*$^{C1041G/+}$ mice (Figure 2, S3, and Videos 1 and 2). The pathology of aneurysm at both the celiac and superior mesenteric arterial branches was characterized by comparable degrees of elastic fragmentation and regeneration, collagen deposition, expansion of the number of cells expressing α-smooth muscle cell actin, and the presence of numerous CD68 positive cells (Figures 2C and S4), when compared to tissues from the same regions of *Fbn1*$^{+/+}$ and *Fbn1*$^{C1041G/+}$ mice infused with saline or *Fbn1*$^{+/+}$ mice infused with AngII.

## DISCUSSION

There is an increasing awareness that *FBN1* variants can lead to a spectrum of aortic pathologies. For example, it has been recognized recently that branch aneurysms are frequent in Marfan Syndrome patients and are associated with vascular risk.[3] This study has demonstrated that infusion of AngII into mice expressing the C1041G variant of *Fbn1* leads to augmented aneurysms in the proximal thoracic aorta, accelerated aortic dissection and rupture, and aortic branch aneurysms.

Our previous studies demonstrated the age-dependent expansion of the ascending aorta in the *Fbn1*$^{C1041G/+}$ has a strong sexual dimorphism, with males showing gradual dilation over the course of a year, whereas females display a rate of increase indistinguishable from that of *Fbn1*$^{+/+}$ mice.[8] These results were replicated in the current study. Sexual dimorphism was also present in some aspects of aortic diseases during AngII infusion. Specifically, there was a much greater incidence of death due to loss of aortic integrity in males compared to females. However, similar to males, female *Fbn1*$^{C1041G/+}$ mice infused with AngII had striking expansion of the ascending aorta. This finding implies that distinct mechanisms underlie aneurysm formation and rupture in *Fbn1*$^{C1041G/+}$ mice, which will need to be further elucidated.

The basis for AngII promoting aortic diseases in a regional manner is unclear. There have been several proposed mechanisms for the regional specificity of aortic disease, with a major emphasis on embryonic origins of smooth muscles cells.[26, 27] It has been established that AngII-induced aortic pathologies are due to stimulation of AT1a receptors.[28] However, deletion of AT1a receptors in aortic smooth muscle cells has not been demonstrated to influence aortic diseases, while its deletion in endothelium has a modest reduction compared to whole body deletions.[29-31] Given that the aortic adventitia is heterogeneous in its composition throughout the length of the vessel, this is a potential basis for this regional heterogeneity. Region-specific cell types include perivascular adipocytes and fibroblasts.[32, 33] Resolving the contributions of adventitial components to aortic pathology will require the development of mice expressing Cre under the control of promoters that specifically target these cell types.

A novel observation in this study is the presence of aortic branch aneurysms predominantly involving the celiac and superior mesenteric arteries in AngII-infused *Fbn1*$^{C1041G/+}$ mice, irrespective of sex. This finding parallels recent clinical reports demonstrating aortic branch aneurysms in patients with Marfan syndrome.[3] Consistent with these observations, the 2022 ACC/AHA guidelines recognize that aortic branch aneurysms are more common than previously appreciated, with an elevated likelihood of requiring aortic surgery.[34] The mechanisms by which AngII preferentially promotes aneurysm formation in the celiac and superior mesenteric arteries of *Fbn1*$^{C1041G/+}$ mice remain unclear. Both arteries supply blood to major gastrointestinal organs, and the superior mesenteric artery functions as a resistant artery critical for regulating regional blood flow and systemic blood pressure. Their unique hemodynamic demands, complex branching geometry, and distinct extracellular matrix architecture may render these two arteries susceptible to the combined effects of *Fbn1* haploinsufficiency and AngII-induced mechanical and inflammatory stress. Given their central roles in abdominal organ perfusion, further exploration is warranted to elucidate how AngII drives aneurysmal development and vascular remodeling in these specific arteries of *Fbn1*$^{C1041G/+}$ mice.

Infusion of AngII at sufficient rates increases systolic blood pressure, which may contribute to aortic pathology. One strategy to determine the role of blood pressure has been to compare pathology in mice infused with either AngII or NE at rates that produce equivalent increases in systolic blood pressure. Using this approach, blood pressure *per se* has not been implicated in the development of atherosclerosis,[35] abdominal aortic aneurysm,[36] or ascending aortic aneurysms.[20] In this study, NE increased systolic blood pressure but failed to replicate the aortic pathologies generated during AngII infusion which implies that increased blood pressure is not a sufficient cause of the disease. AngII increases systolic blood pressure, which may contribute to the aortic pathology.

In summary, this study demonstrates that AngII infusion in *Fbn1*$^{C1041G/+}$ mice augments aneurysmal disease in the distal thoracic aorta and induces previously undocumented pathologies, including aortic rupture and aortic branch aneurysms. This model provides a valuable platform to perform subsequent studies to define the mechanisms of these diverse pathologies.

## Acknowledgments

These studies were facilitated by the University of Kentucky Light Microscopy Core (RRID:SCR 026405) and the Magnetic Resonance and Spectroscopy Core (RRID:SCR 026383).

## Sources of funding

This research work is supported by the National Heart, Lung, and Blood Institute of the National Institutes of Health (R35HL155649, K01HL149984), a Merit award from the American Heart Association (23MERIT1036341), and a Leducq Foundation Network of Excellence (22CVD03).

## Disclosures

The authors have no conflicts of interest.

## FIGURE LEGENDS

**Figure 1.** **AngII Infusion Augmented Ascending Aortic Aneurysm in *Fbn1*$^{C1041G/+}$ mice and Induced Aortic Rupture.** Ten-14-week-old male and female *Fbn1*$^{+/+}$ and *Fbn1*$^{C1041G/+}$ mice were infused with either saline or AngII for 28 days. Survival curves and cause of death in male (**A-B**) and female (**C-D**) mice. P values (**A, C**) were determined by Log-Rank analysis. Representative in situ images of the thoracic aorta and maximal diameters of ascending aortas in male (**E-F**) and female (**G-H**) mice. Representative ex vivo images of the entire aorta in male (**I**) and female (**J**) mice. Aortic length measured from the left subclavian branch to the iliac bifurcation in male (**K**) and female (**L**) mice. Data in (**F**, **H**, **L**) were analyzed using Mann-Whitney Rank Sum test, and data in (**F**, **K**) were analyzed using Student's t-test.

**Figure 2.** **AngII Promoted Development of Aortic Branch Aneurysms in *Fbn1*$^{C1041G/+}$ Mice.** Ten-14-week-old male *Fbn1*$^{+/+}$ and *Fbn1*$^{C1041G/+}$ mice were infused with either saline or AngII for 28 days. (**A**) Representative microCT images of the entire aorta and the abdominal aorta. (**B**) Maximum diameters of the aortic branch points for celiac, superior mesenteric, right renal, and left renal arteries measured using microCT images. Statistical analysis was performed using Mann-Whitney Rank Sum test. (**C**) Tissue sections of the celiac and superior mesenteric aortic branches were stained with Verhoeff's iron hematoxylin, picrosirius red/methyl green, and immunostained for α-smooth muscle actin and CD68, respectively.

## Supplemental Figure Legends

**Figure S1.** **AngII Infusion Led to Rapid Formation of Thoracic Aortic Dissection.** In situ images of the ascending and descending thoracic aorta of male *Fbn1*$^{+/+}$ and *Fbn1*$^{C1041G/+}$ mice infused with AngII for 3 days.

**Figure S2.** **NE Did not Promote Evident Aortic Pathologies in *Fbn1*$^{+/+}$ and *Fbn1*$^{C1041G/+}$ Mice.** Ten-14-week-old male and female *Fbn1*$^{+/+}$ and *Fbn1*$^{C1041G/+}$ mice were infused with either vehicle (0.2% wt/vol ascorbic acid dissolved in saline) or NE for 28 days. Systolic blood pressure measurements in male (**A**) and female (**B**) mice. Survival curves for male (**C**) and female (**D**) mice. Representative in situ images of the thoracic aorta and maximal diameters of ascending aortas in male (**E-F**) and female (**G-H**) mice. Representative ex vivo images of the entire aorta in male (**I**) and female (**K**) mice. Aortic length measured from the left subclavian branch to the iliac bifurcation in male (**J**) and female (**L**) mice. Data in (**A**, **F**, **H**, **J**, and *Fbn1*$^{+/+}$ mice in **B** and **L**) were analyzed using Mann-Whitney Rank Sum test, and data in *Fbn1*$^{C1041G/+}$ mice (**B**, **L**) were analyzed using Student's t-test.

**Figure S3.** **AngII Infusion Promoted Development of Abdominal Aortic Brach Aneurysms in Female *Fbn1*$^{C1041G/+}$ Mice.** Ten-14-week-old female *Fbn1*$^{+/+}$ and *Fbn1*$^{C1041G/+}$ mice were infused with either saline or AngII for 28 days. (**A**) Representative microCT images of the entire aorta and the abdominal aorta. (**B**) Maximum diameters of the aortic branch points for celiac, superior mesenteric, right renal, and left renal arteries measured using microCT images. Statistical analysis was performed using Mann-Whitney Rank Sum test.

**Figure S4.** **Pathological Characterization of the Celiac and Superior Mesenteric Arteries.** Ten-14-week-old female *Fbn1*$^{+/+}$ and *Fbn1*$^{C1041G/+}$ mice were infused with either saline or AngII for 28 days. Arterial tissue sections were stained with Verhoeff's iron hematoxylin, picrosirius red/methyl green, and immunostained for α-smooth muscle actin and CD68, respectively.

**Video 1.** **MicroCT Images of Saline-infused *Fbn1*$^{+/+}$ and *Fbn1*$^{C1041G/+}$ Male Mice.**

**Video 2.** **MicroCT Images of AngII-infused *Fbn1*$^{+/+}$ and *Fbn1*$^{C1041G/+}$ Male Mice.**

Figure 1

A
Fbn1 +/+
Fbn1C1041G/+
Saline
AngII
B
Celiac
Superior Mesenteric
Right Renal
Left Renal
Fbn1+/+
Fbn1C1041G /+
Fbn1C1041G/+
P=0.73
P=0.016
P>0.99
P=0.032
P=0.41
P=0.11
P=0.11
P=0.063
Maximal Diameter (mm)
2.0
1.5
1.0
0.5
0.0
Saline AngII Saline AngII
C
Celiac
SMA
Verhoeff's
Picro.Red/Meth.Green
α-SMA
CD68
200 μm

Figure 2
